# Supplementary material for: Evidence of megathrust earthquakes and seismic supercycles in subtropical Japan from millennia-old coral microatolls
Source: Nat Commun. 2026 Feb 10;17:1398. doi: 10.1038/s41467-025-67724-2 (PMC12891547; doi:10.1038/s41467-025-67724-2)
Supplement: Supplementary file 1 — Supplementary Information [file 41467_2025_67724_MOESM1_ESM.pdf]

Supplementary Information for:

Evidence of megathrust earthquakes and seismic supercycles in subtropical Japan from millennia-old coral microatolls

By Sophie Debaecker, Nathalie Feuillet, Kenji Satake, Kohki Sowa, Masaki Yamada, Tetsuro Sato, Mamoru Nakamura, Atsushi Watanabe, Ayaka Saiki, Jean-Marie Saurel, Giovanni Occhipinti, Tsai-Luen Yu and Chuan-Chou Shen.

Contact: debaecker.sophie@gmail.com

Contents

S1. Site and Data presentation

S2. Coral analyses

S3. Elastic modelling

Supplementary Information S1: Site and Data presentation

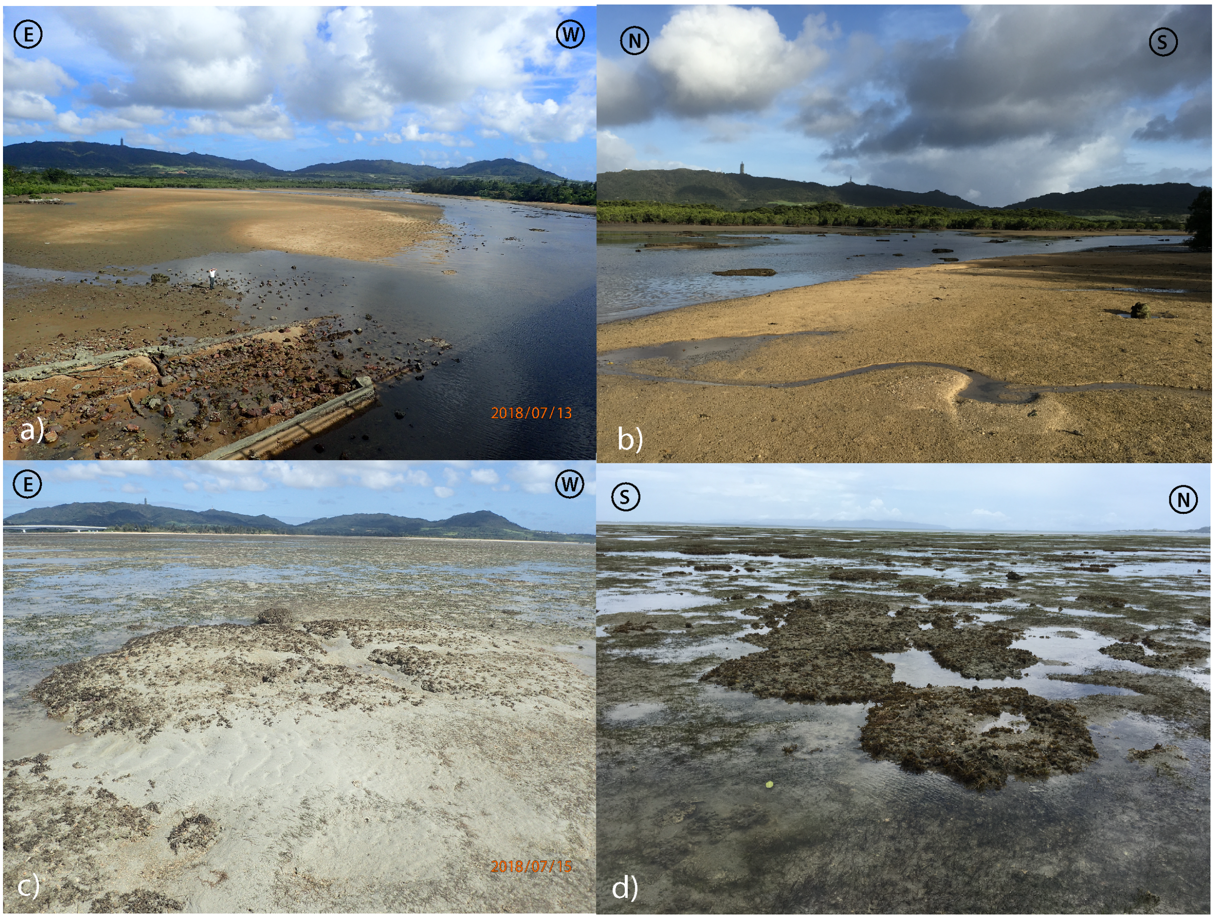

Figure S1. Photographs of Nagura site a) and b) in the mangrove area, and c) and d) in the bay area.

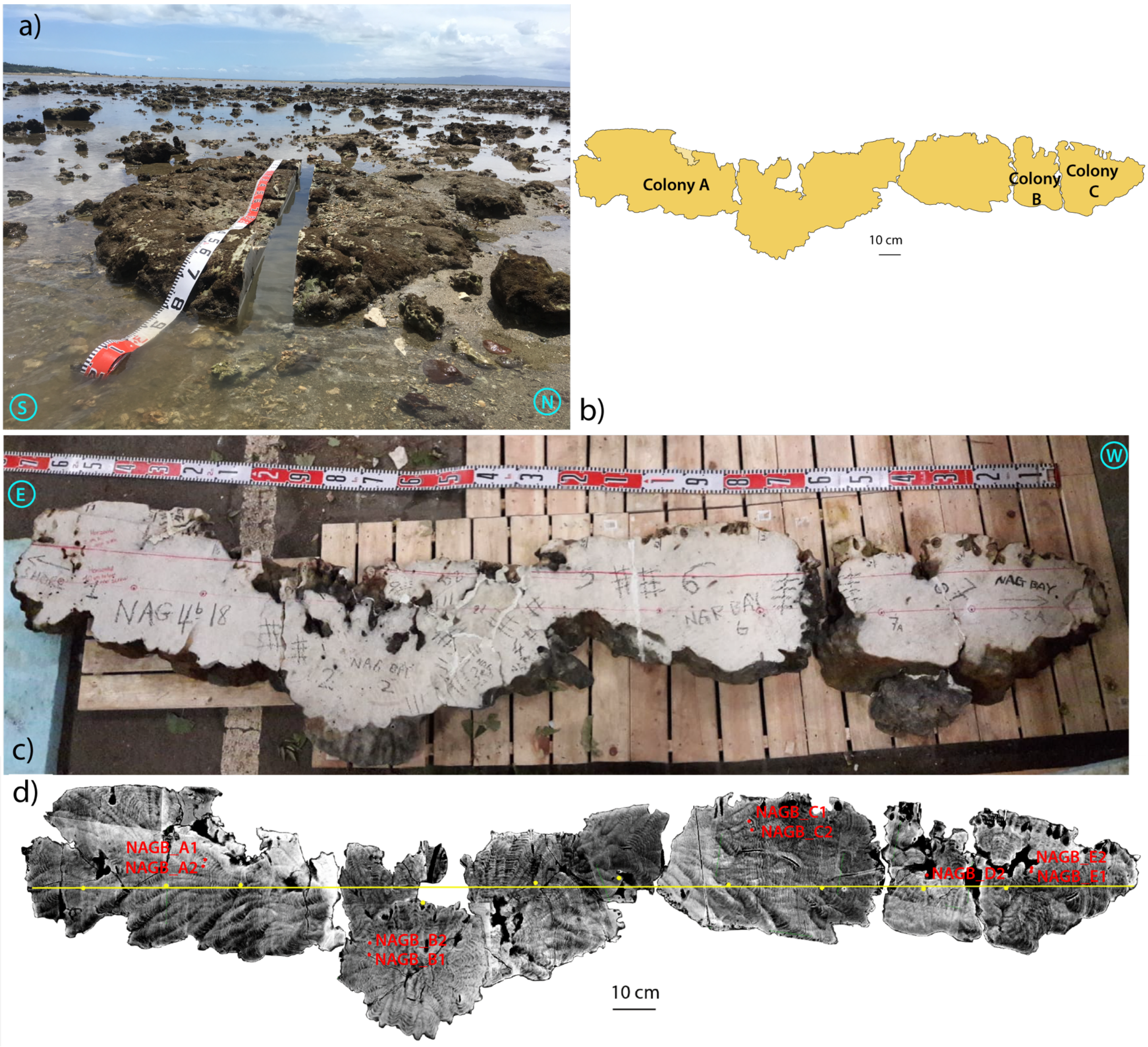

Figure S2. NAG_B microatoll. a) Photograph of NAG_B coral after slicing. b) Schematic draw of the coral with its different colonies. c) Photograph of the 10 cm-thick slice. d) Xray mosaic image of NAG_B. Yellow line the original horizontal position before slicing, retrieved from elevation measurements of screws along the slice (yellow points). Red points show microcores samples for geochemical analysis.

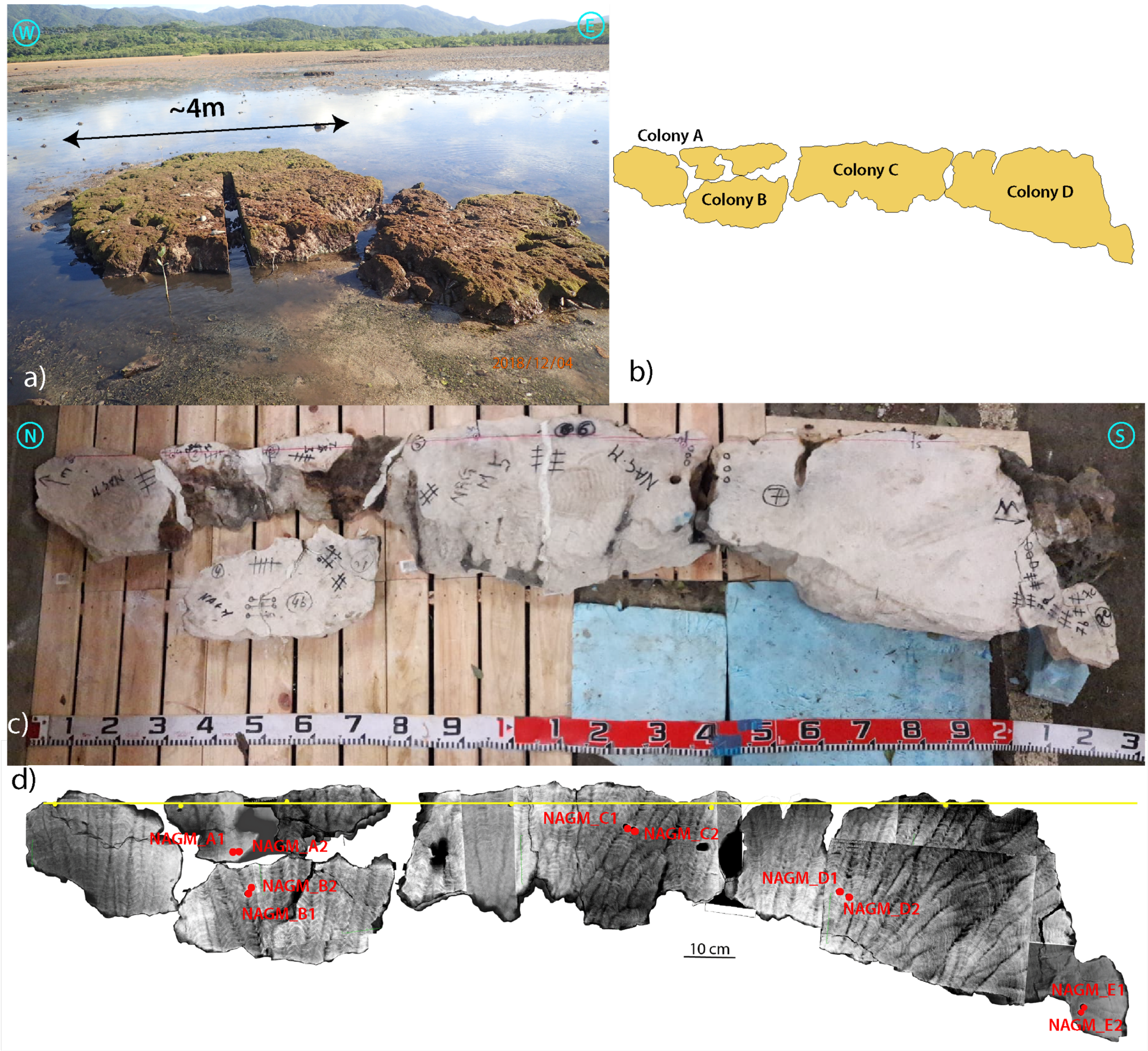

Figure S3. NAG_M microatoll. a) Photograph of NAG_M coral after slicing. b) Schematic draw of the coral with its different colonies. c) Photograph of the 10 cm-thick slice. d) Xray mosaic image of NAG_M. Yellow line and points, and red points as in figure S2.

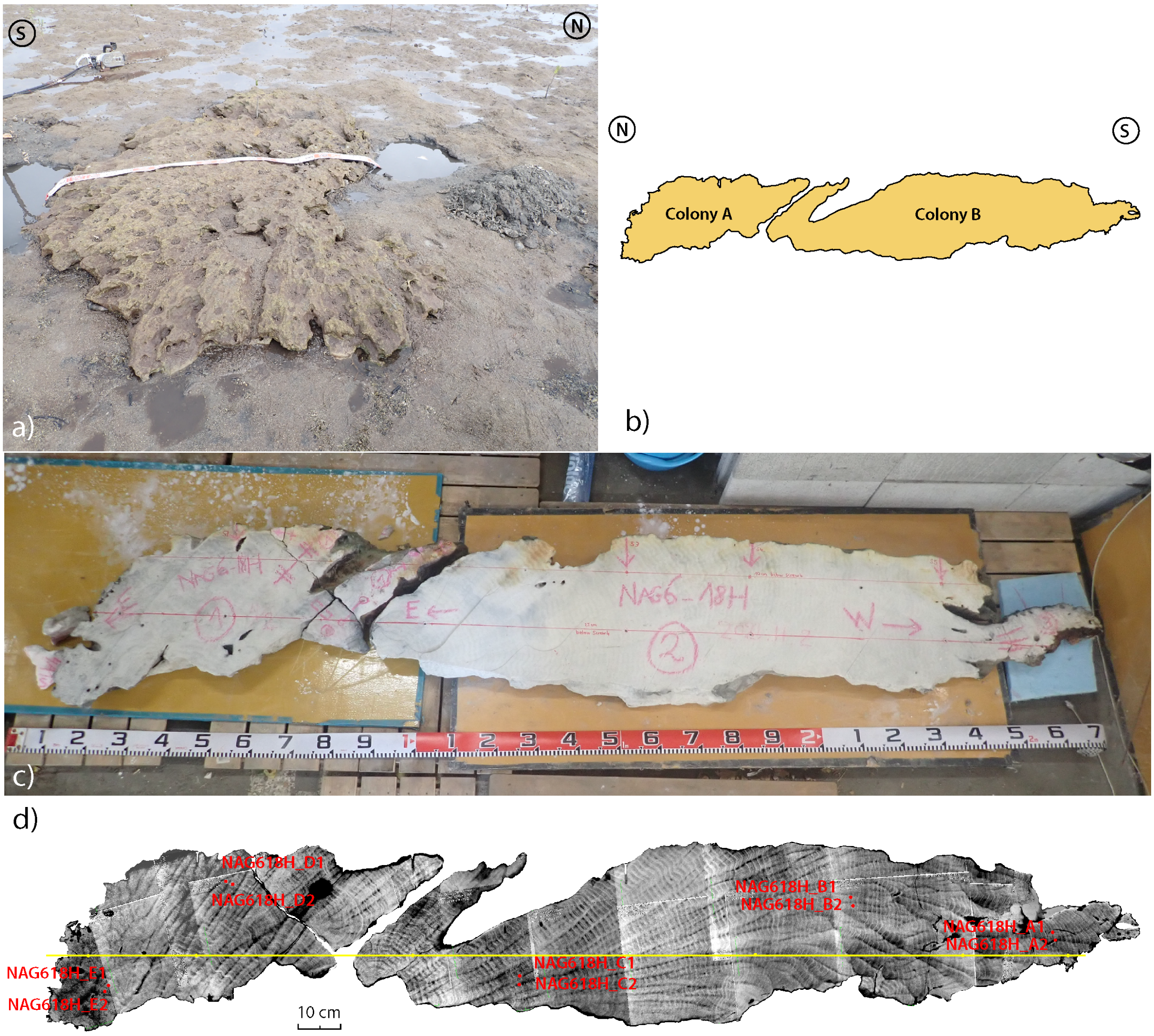

Figure S4. NAG6_18H microatoll. a) Photograph of NAG6_18H coral before slicing. b) Schematic draw of the coral with its different colonies. c) Photograph of the 10 cm-thick slice. d) Xray mosaic image of NAG6_18H. Yellow line and points, and red points as in figure S2.

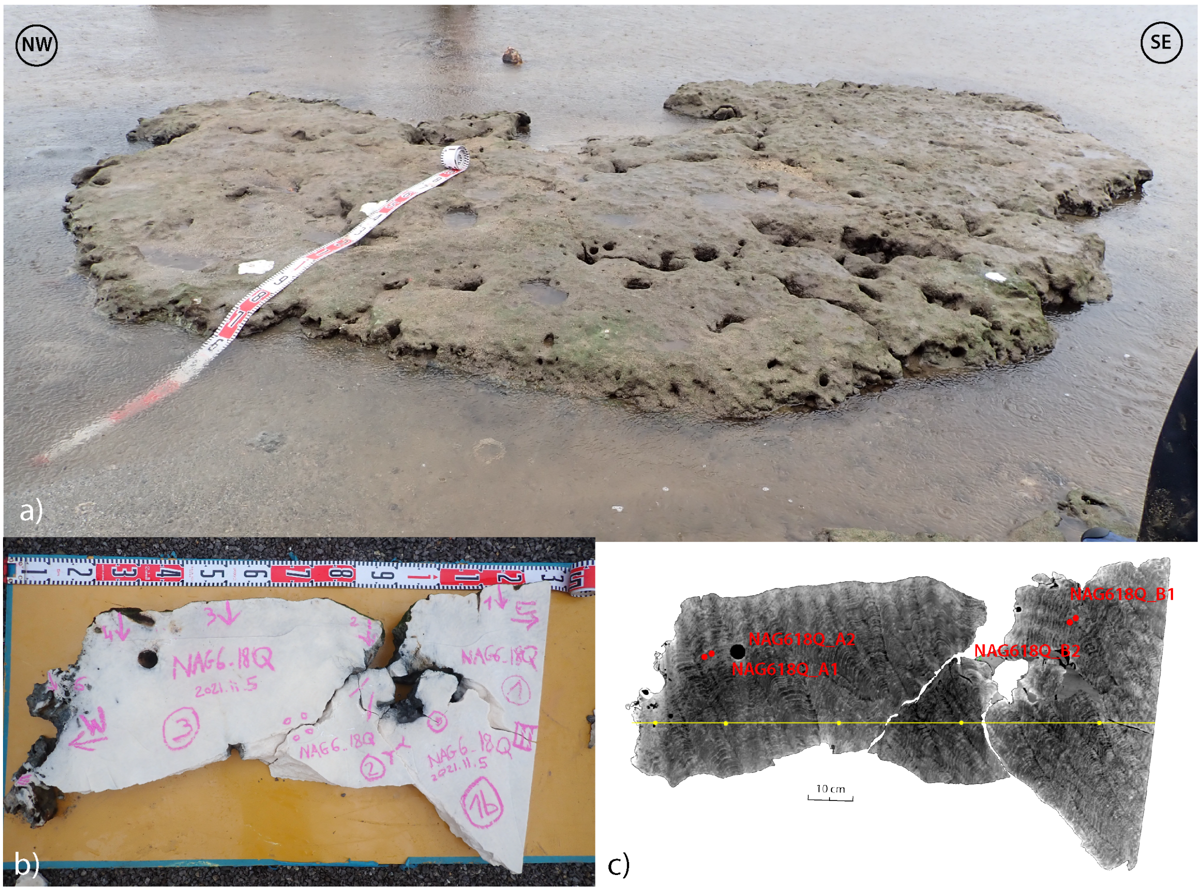

Figure S5. NAG6_18Q microatoll. a) Photograph of NAG6_18Q coral before slicing. b) Photograph of the 10 cm-thick slice. c) Xray mosaic image of NAG6_18Q. Yellow line and points, and red points as in figure S2.

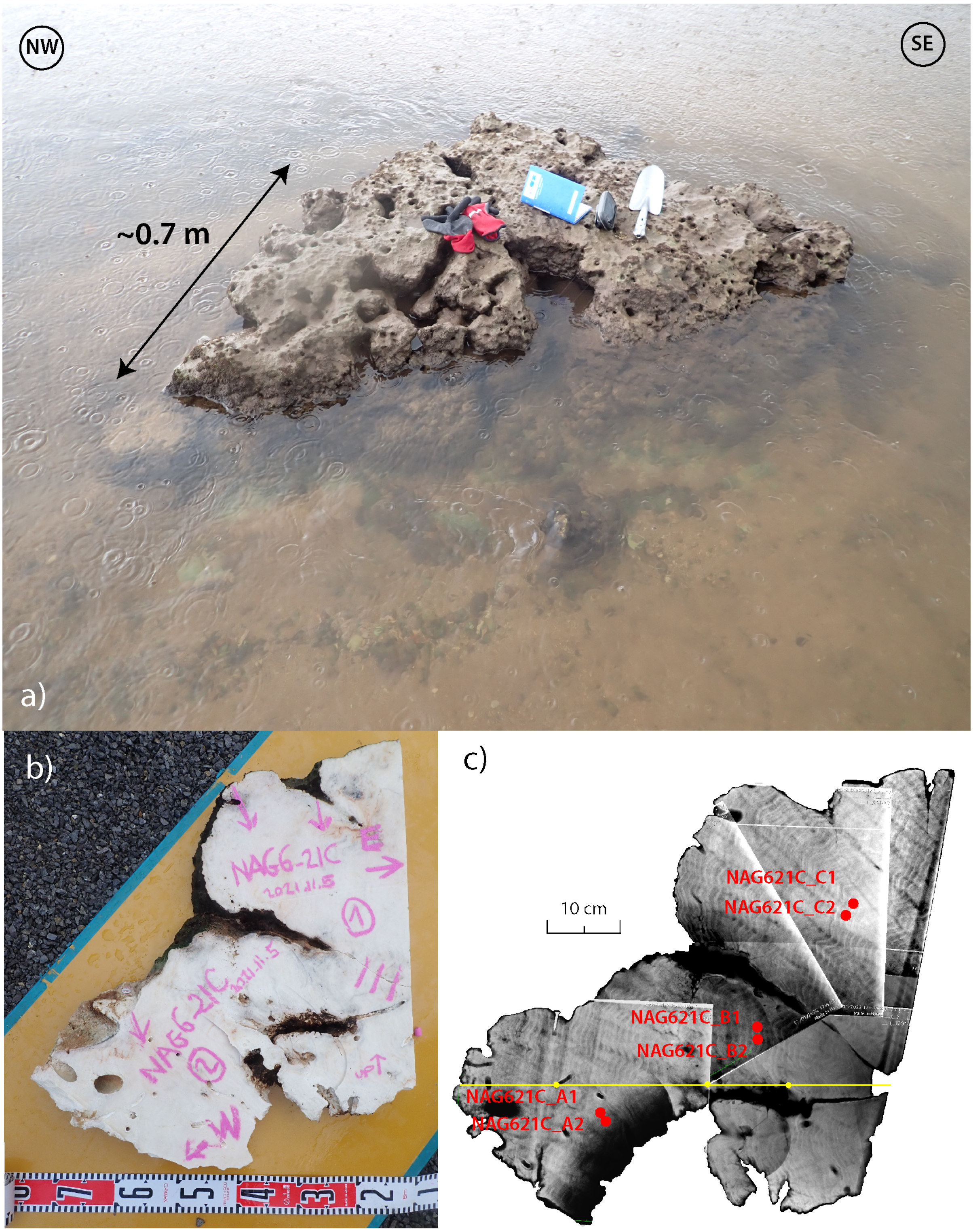

Figure S6. NAG6_21C microatoll. a) Photograph of NAG6_21C coral before slicing. b) Photograph of the 10 cm-thick slice. c) Xray mosaic image of NAG6_21C. Yellow line and points, and red points as in figure S2.

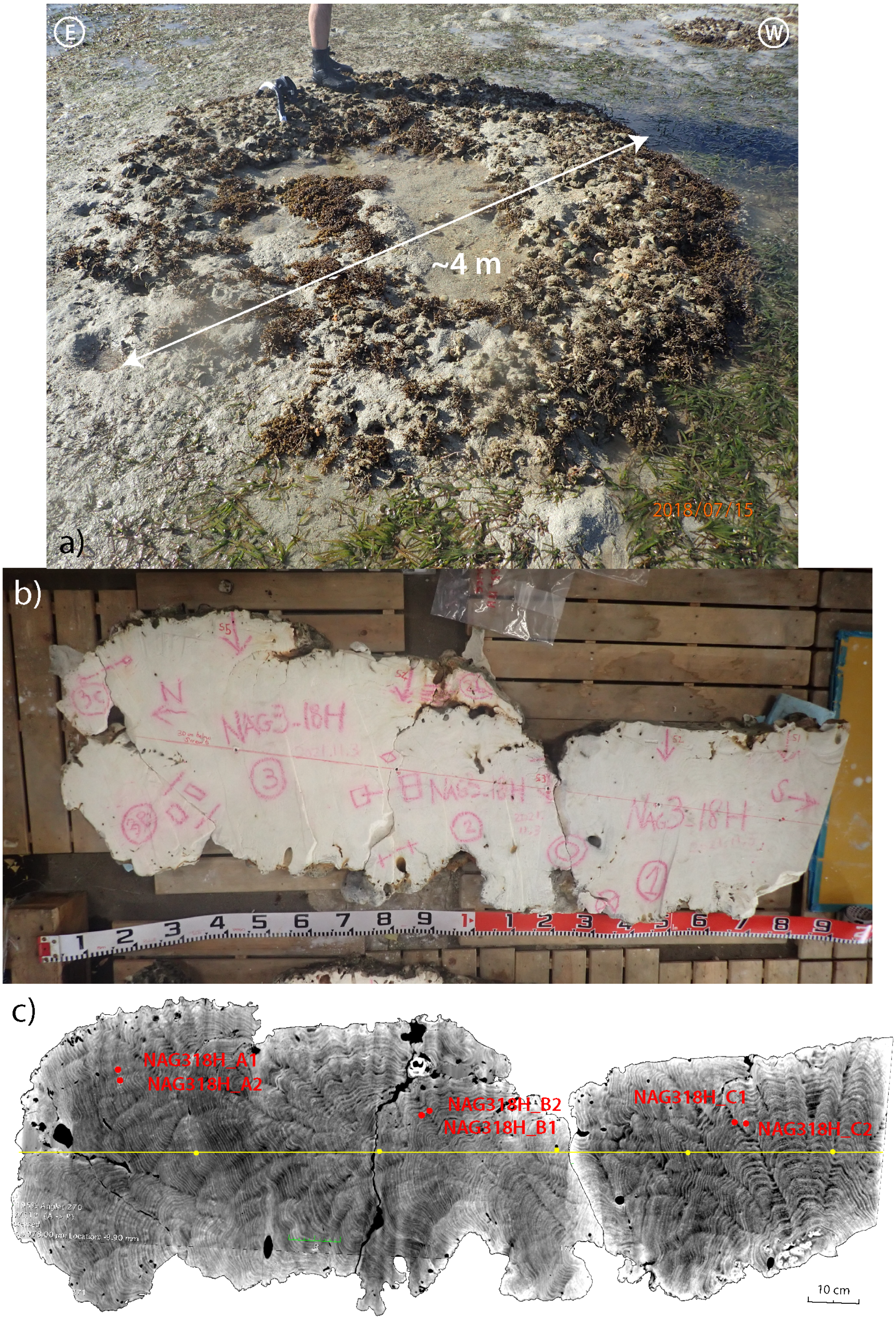

Figure S7. NAG3_18H microatoll. a) Photograph of NAG3_18H coral before slicing. b) Photograph of the 10 cm-thick slice. c) Xray mosaic image of NAG3_18H. Yellow line and points, and red points as in figure S2.

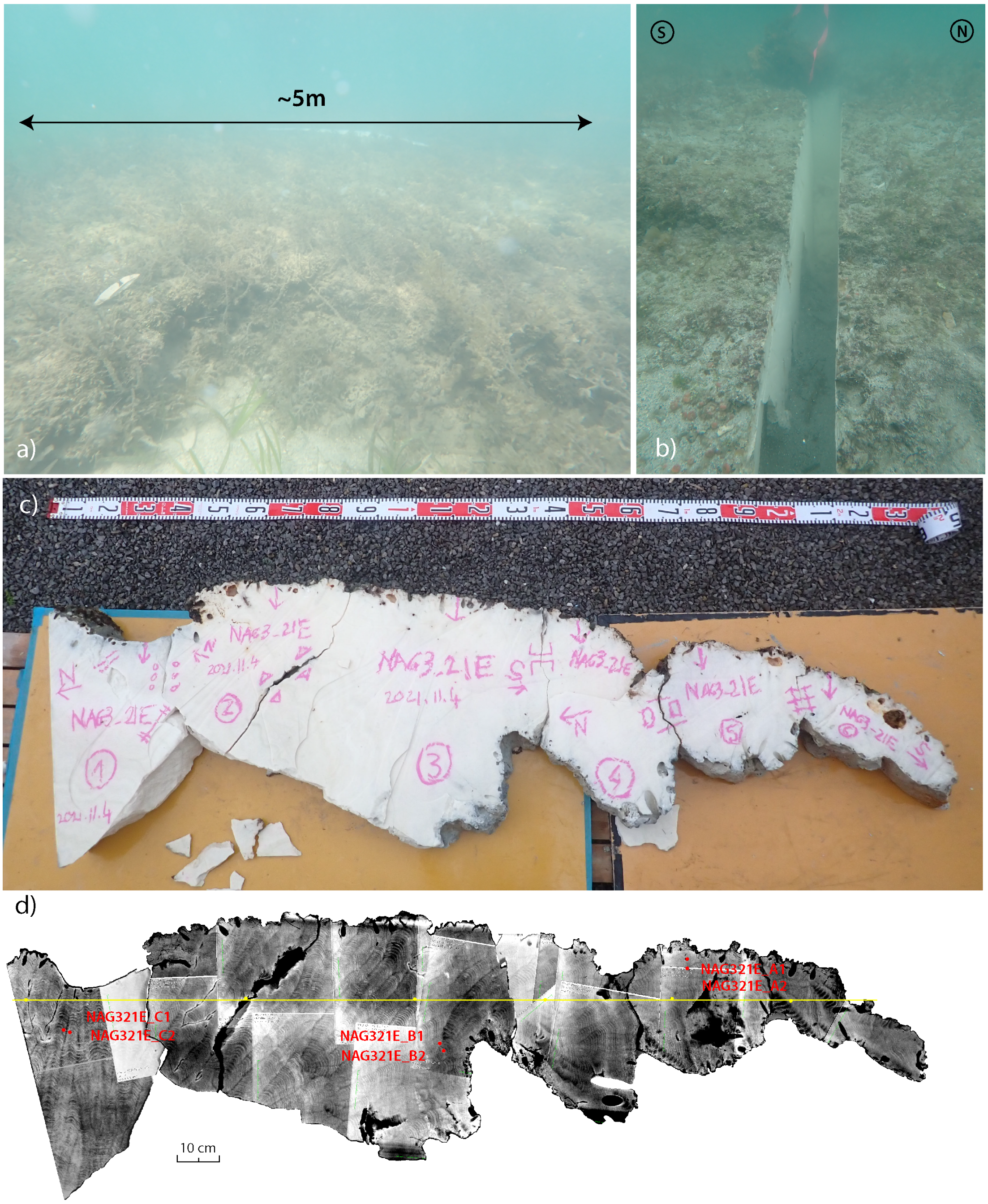

Figure S8. NAG3_21E microatoll. a) Photograph of NAG3_21E coral before slicing. b) Photograph of NAG3_21E coral after slicing. c) Photograph of the 10 cm-thick slice.d) Xray mosaic image of NAG3_21E. Yellow line and points, and red points as in figure S2.

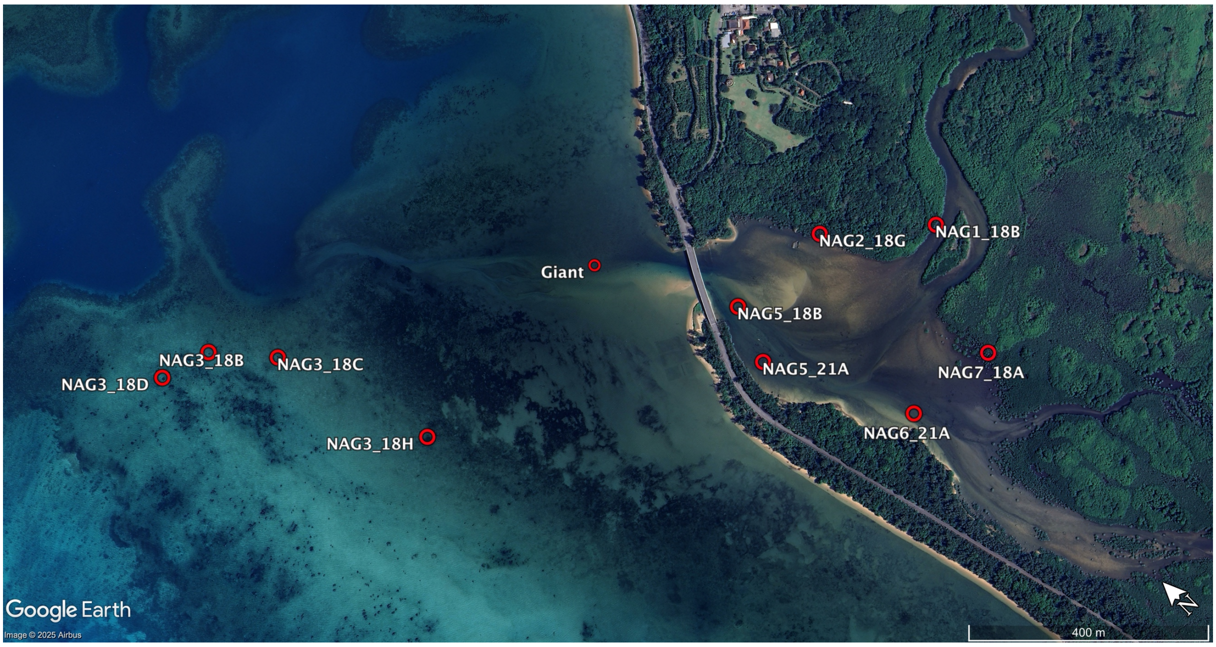

Figure S9. Location of the samples of Nagura site for U/Th dating, superimposed on Google Earth screenshot (© 2025 Maxar Technologies, CNES / Airbus, Google Earth).

Table S1: Samples in Nagura site

**Sample**	Location	Diameter (m)	Thickness (cm)	Particularity
**NAG1_18B**	Mangrove	3	40	Incomplete coral, several rings
**NAG2_18G**	Mangrove	3.5	70	Incomplete coral, several rings
**NAG5_18B**	Mangrove	2	30	Roundshape, pretty flat
**NAG7_18A**	Mangrove	4.5	30	Three main rings, bioturbated
**NAG6_21A**	Mangrove			Partially buried with a lower lobe
**NAG5_21A**	Mangrove			Incomplete coral with a possible lower lobe
**GIANT**	Bay	>10	>30	Buried. Very large microatoll which may be composed of several colonies
**NAG3_18B**	Bay	2.5	>20	Buried, slightly cupshape
**NAG3_18C**	Bay	4	>30	Buried, bioturbation
**NAG3_18D**	Bay	3	>20	Buried, cupshape
**NAG3_18H**	Bay	2.5	>40	Buried, clear cupshape

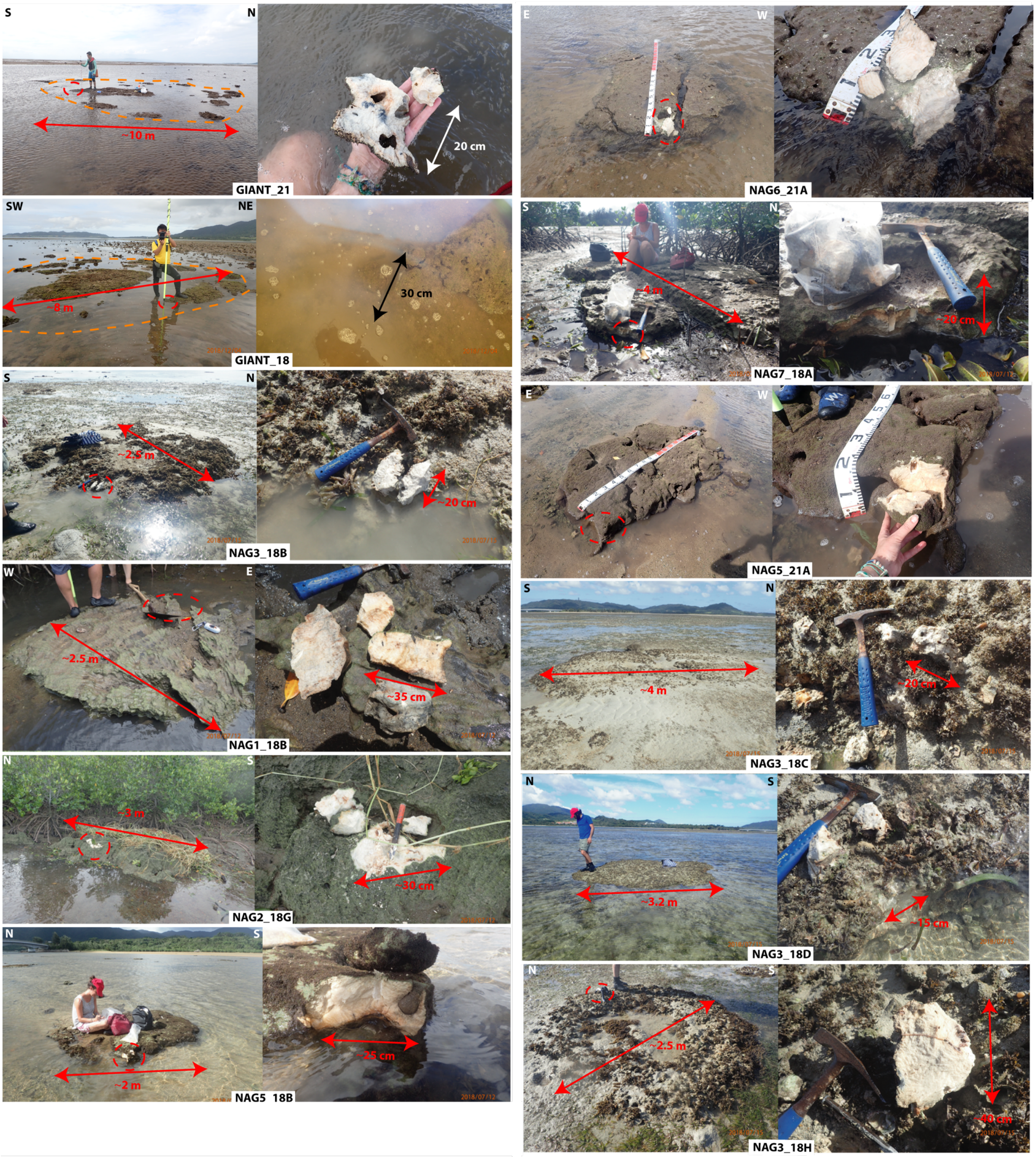

Fig. S10: Samples in Nagura site. Left is the main coral we sampled and right is the sample we selected for U/Th datation. Red arrows indicate the scale but do not necessarily correspond to the coral diameter, as their shape was not always clearly round.

Table S2. U-Th dating results

Sample ID	Weight g	^{238}U 10^{-6}g/g [a]		^{232}Th 10^{-9}g/g		$\delta^{234}U$ measured[a]		[$^{230}Th/^{238}U$] activity[c]		$^{230}Th/^{232}Th$ atomic (x 10^{-6})		Age (kyr ago) uncorrected		Age (ka) corrected [c,d]		$\delta^{234}U_{initial}$ corrected[b]		Age (year B.C.)
**Slices**																		
**NAG_M**																		
**NAGM_A1**	0.2095	2.7808	± 0.0045	1.7163	± 0.0052	137.4	± 2.2	0.043556	± 0.00022	1163.5	± 6.5	4.255	± 0.023	4.172	± 0.024	139.1	± 2.2	2222±24
**NAGM_C1**	0.2192	2.7190	± 0.0023	0.4411	± 0.0022	143.8	± 1.5	0.043696	± 0.00010	4441.3	± 25	4.244	± 0.012	4.170	± 0.012	145.5	± 1.5	2220±12
**NAGM_D1**	0.2055	2.5358	± 0.0023	0.4902	± 0.0024	143.4	± 1.6	0.044016	± 0.00014	3754.4	± 21	4.277	± 0.015	4.202	± 0.015	145.2	± 1.6	2252±15
**NAGM_E1**	0.173	2.7236	± 0.0045	0.2527	± 0.0027	141.7	± 2.3	0.044167	± 0.00012	7848.7	± 86	4.299	± 0.015	4.228	± 0.015	143.5	± 2.3	2278±15
**NAG_B**																		
**NAGB_A1**	0.1931	2.7581	± 0.0092	1.0924	± 0.0058	147.3	± 4.6	0.024234	± 0.00014	1008.8	± 7.3	2.327	± 0.017	2.249	± 0.017	148.2	± 4.6	299±17
**NAGB_B2**	0.1816	2.5424	± 0.010	2.5103	± 0.010	141.8	± 5.1	0.025094	± 0.00017	419.0	± 2.9	2.422	± 0.020	2.331	± 0.023	142.8	± 5.1	381±23
**NAGB_C1**	0.1762	2.6163	± 0.0070	1.7205	± 0.0057	146.4	± 3.7	0.024161	± 0.00015	605.8	± 3.9	2.322	± 0.016	2.238	± 0.018	147.4	± 3.7	288±18
**NAGB_D1**	0.1546	2.6075	± 0.010	4.7483	± 0.025	142.2	± 5.1	0.023282	± 0.00022	210.8	± 2.1	2.245	± 0.023	2.134	± 0.031	143.1	± 5.2	184±31
**NAG6_18Q**																		
**NAG618Q_A1**	0.3918	3.0105	± 0.0037	0.9018	± 0.0016	147.0	± 1.6	0.045540	± 0.00010	2506.7	± 6.6	4.414	± 0.012	4.335	± 0.013	148.9	± 1.6	2385±13
**NAG618Q_B1**	0.2141	3.0263	± 0.0029	0.6894	± 0.0023	145.6	± 1.4	0.045800	± 0.000086	3315.2	± 12	4.446	± 0.010	4.368	± 0.011	147.4	± 1.4	2418±11
**NAG6_21C**																		
**NAG621C_A1**	0.2943	2.5330	± 0.0027	1.2269	± 0.0022	144.0	± 1.5	0.045687	± 0.00011	1555.1	± 4.4	4.440	± 0.013	4.357	± 0.014	145.9	± 1.5	2407±14
**NAG621C_C1**	0.2765	2.6446	± 0.0030	0.2487	± 0.0017	147.4	± 1.6	0.046189	± 0.000075	8097.7	± 56	4.477	± 0.010	4.402	± 0.010	149.3	± 1.6	2452±10
**NAG3_21E**																		
**NAG321E_A1**	0.4842	2.7635	± 0.0027	56.0223	± 0.25	148.7	± 1.6	0.027977	± 0.00046	22.8	± 0.39	2.687	± 0.045	2.150	± 0.238	149.6	± 1.7	198±238
**NAG321E_B1**	0.4254	2.9352	± 0.0033	0.4767	± 0.0012	148.1	± 1.5	0.024067	± 0.000054	2443.4	± 7.6	2.309	± 0.006	2.233	± 0.006	149.0	± 1.5	283±6
**NAG321E_C1**	0.4561	2.7664	± 0.0033	0.2186	± 0.0010	148.4	± 1.6	0.024585	± 0.000047	5130.0	± 26	2.359	± 0.006	2.285	± 0.006	149.3	± 1.6	335±6
**NAG3_18H**																		
**NAG318H_A2**	0.5857	2.8716	± 0.0029	3.0647	± 0.0044	147.7	± 1.6	0.025588	± 0.00011	395.3	± 1.8	2.457	± 0.012	2.361	± 0.017	148.8	± 1.6	411±17
**NAG318H_C1**	0.4871	2.7860	± 0.0017	1.9848	± 0.0020	147.6	± 1.4	0.027032	± 0.000091	625.6	± 2.2	2.5981	± 0.009	2.509	± 0.012	148.7	± 1.4	559±12
**NAG6_18H**																		
**NAG618H_A2**	0.5327	2.8239	± 0.0032	22.6119	± 0.061	146.9	± 1.6	0.044334	± 0.00043	91.3	± 0.91	4.295	± 0.043	4.040	± 0.102	148.6	± 1.7	2089±102
**NAG618H_C2**	0.2921	2.6138	± 0.0024	2.2882	± 0.0033	145.0	± 1.5	0.044141	± 0.00015	831.4	± 2.9	4.284	± 0.016	4.191	± 0.019	146.8	± 1.5	2241±19
**NAG618H_D2**	0.335	2.7879	± 0.0027	6.9354	± 0.013	146.7	± 1.4	0.045177	± 0.00021	299.4	± 1.5	4.380	± 0.022	4.250	± 0.036	148.5	± 1.4	2300±36
**NAG618H_E1**	0.2935	2.7787	± 0.0029	3.0121	± 0.0044	147.4	± 1.5	0.045130	± 0.00018	686.4	± 2.8	4.372	± 0.019	4.275	± 0.022	149.2	± 1.5	2325±22

Sample ID	Weight g	^{238}U 10^{-6}g/g [a]		^{232}Th 10^{-9}g/g		$\delta^{234}U$ measured[a]		[$^{230}Th/^{238}U$] activity[c]		$^{230}Th/^{232}Th$ atomic (x 10^{-6})		Age (kyr ago) uncorrected		Age (ka) corrected [c,d]		$\delta^{234}U_{initial}$ corrected[b]		Age (year B.C.)
**Block samples**																		
**NAG3_18C**	0.1662	2.6860	± 0.0023	45.8423	± 0.44	144.4	± 1.3	0.031488	± 0.00079	30.4	± 0.81	3.041	± 0.077	2.580	± 0.212	145.5	± 1.4	627±212
**NAG3_18H**	0.1568	2.8088	± 0.0026	8.7958	± 0.035	145.8	± 1.4	0.025455	± 0.00028	134.0	± 1.5	2.449	± 0.027	2.308	± 0.045	146.8	± 1.4	358±45
**NAG3_18D**	0.1272	2.4555	± 0.0022	12.7777	± 0.060	148.3	± 1.6	0.023940	± 0.00034	75.9	± 1.1	2.296	± 0.033	2.108	± 0.068	149.2	± 1.6	158±68
**NAG2_18G**	0.1624	2.8568	± 0.0026	6.5429	± 0.019	146.3	± 1.4	0.050680	± 0.00029	364.8	± 2.3	4.927	± 0.030	4.805	± 0.040	148.3	± 1.4	2855±40
**NAG7_18A**	0.1647	2.7810	± 0.0024	6.3558	± 0.022	146.1	± 1.5	0.046918	± 0.00031	338.5	± 2.5	4.554	± 0.032	4.432	± 0.041	148.0	± 1.6	2482±41
**NAG1_18B**	0.185	2.8066	± 0.0030	15.9448	± 0.070	143.1	± 1.6	0.050164	± 0.00046	145.6	± 1.5	4.889	± 0.047	4.689	± 0.080	145.0	± 1.6	2739±80
**NAG3_18B**	0.1837	2.5307	± 0.0029	20.9807	± 0.18	144.9	± 1.6	0.025587	± 0.00065	50.9	± 1.4	2.464	± 0.064	2.200	± 0.115	145.8	± 1.6	253±115
**NAG5_18B**	0.1627	2.6917	± 0.0027	12.0379	± 0.054	141.3	± 1.7	0.042074	± 0.00040	155.1	± 1.6	4.093	± 0.040	3.920	± 0.065	142.9	± 1.7	1970±65
**NAG5_21A**	0.7002	2.1483	± 0.0022	60.9142	± 0.82	146.0	± 1.4	0.049457	± 0.00021	28.8	± 0.40	4.806	± 0.021	4.080	± 0.330	147.7	± 1.4	2128±330
**NAG6_21A**	0.361	2.5837	± 0.0017	16.8190	± 0.039	143.4	± 1.3	0.045506	± 0.00035	115.3	± 0.91	4.425	± 0.035	4.202	± 0.083	145.1	± 1.3	2252±83
**GIANT_21**	0.2054	2.7644	± 0.0034	2.7591	± 0.0065	140.8	± 1.7	0.034716	± 0.00020	573.5	± 3.5	3.368	± 0.020	3.274	± 0.023	142.1	± 1.7	1324±23
**GIANT_18**	0.3241	3.6542	± 0.0024	1.2076	± 0.0017	146.0	± 1.3	0.024930	± 0.000063	1243.9	± 3.5	2.397	± 0.007	2.317	± 0.008	147.0	± 1.3	367±8

Analytical errors are 2σ of the mean.

[a] $[^{238}U] = [^{235}U] \times 137.77\ (\pm 0.11‰)$[1] ; $\delta^{234}U = ([^{234}U/^{238}U]_{activity} - 1) \times 1000$.

[b] $\delta^{234}U_{initial}$ corrected was calculated based on ^{230}Th age (T), i.e., $\delta^{234}U_{initial} = \delta^{234}U_{measured} \times e^{\lambda_{234} \times T}$, and T is corrected age.

[c] $[^{230}Th/^{238}U]_{activity} = 1 - e^{-\lambda_{230} T} + (\delta^{234}U_{measured}/1000)[\lambda_{230}/(\lambda_{230} - \lambda_{234})](1 - e^{-(\lambda_{230} - \lambda_{234}) T})$, where T is the age.

Decay constants used are available in Cheng et al.[2].

[d] Age corrections[3], relative to AD 1950, were calculated using an estimated atomic $^{230}Th/^{232}Th$ ratio of 4 ($\pm$ 2) x 10^{-6}.

Table S3. Altitude and age of plateaus identified in Nagura site. Ages of the undated plateaus are bounded by those of the plateau just higher or lower in elevation. * Plateaus 22 and 23 correspond to the two deepest corals observed by Yamaguchi[4]. Estimations on the elevation uncertainty are detailed in the Method section.

Plateau number	Altitude (m)	U/Th Ages (year B.C. with uncertainties)	Inferred age	Coral names
1	0.63±0.09	2855±40 BC		NAG2_18G
2	0.46±0.05		2668±227 BC	NAG2_18A, NAG2_18B
3	0.38±0.04	2482±41 BC		NAG7_18A
				NAG2_18F, NAG2_18C, NAG6_18G
4	0.31±0.05		2553±266 BC	NAG6_18F, NAG6_18M, NAG7_18B
5	0.24±0.04		2553±266 BC	NAG2_18D, NAG6_18C, NAG6_18B, NAG6_18E, NAG2_18E
6	0.18±0.05		2553±266 BC	NAG6_18H, NAG6_18D, NAG5_18G
7	0.11±0.04	2739±80 BC		NAG1_18B
				NAG6_18N, NAG1_18A, NAG5_21D
8	0.03±0.03		2318±190 BC	NAG5_18E, NAG6_18K, NAG6_18A, NAG_M, NAG6_18H, NAG6_18Q, NAG6_18U, NAG6_18L, NAG6_18P, NAG5_18C
9	-0.04±0.05	2252±83 BC		NAG6_21A
				NAG6_21C, NAG6_18V
10	-0.15±0.03	2128±330 BC		NAG5_21A
				NAG5_18F, NAG5_18H, NAG_B, NAG5_21B, NAG5_21C, NAG4_21D
11	-0.25±0.03	1970±65 BC		NAG5_18B
				NAG4_18G, NAG6_18A, NAG_M, NAG6_21B
12	-0.33±0.04	367±8 BC		GIANT
		1324±23 BC		GIANT
				NAG4_18H, NAG6_21A
13	-0.39±0.04		881±466 BC	NAG6_21C, GIANT, C19, C20, C21, C22
14	-0.46±0.05		881±466 BC	C16, C17, C18
15	-0.51±0.03		881±466 BC	NAG3_18G, NAG3_18H, NAG3_21B, NAG3_21C, GIANT, C15
16	-0.59±0.03	627±212 BC		NAG3_18C
				NAG3_18A, NAG3_18H, NAG3_21A, NAG3_21C, NAG3_21D, C13, C14
17	-0.71±0.03	253±115 BC		NAG3_18B
				NAG3_18F, NAG3_21A, NAG3_21D, NAG3_21F, C10
18	-0.82±0.05		403±483 BC	NAG3_21A, NAG3_21E
19	-0.92±0.09		403±483 BC	NAG3_21D
20	-0.99±0.05	2021 AD		Living
				NAG3_18E, NAG3_21E
21	-1.27±0.09		1520±430 AD	C11
22	-2.00±0.50	1157±92* AD		YAMA_2
23	-1.80±0.50	790±97* AD		YAMA_1

Supplementary Information S2: Coral analyses

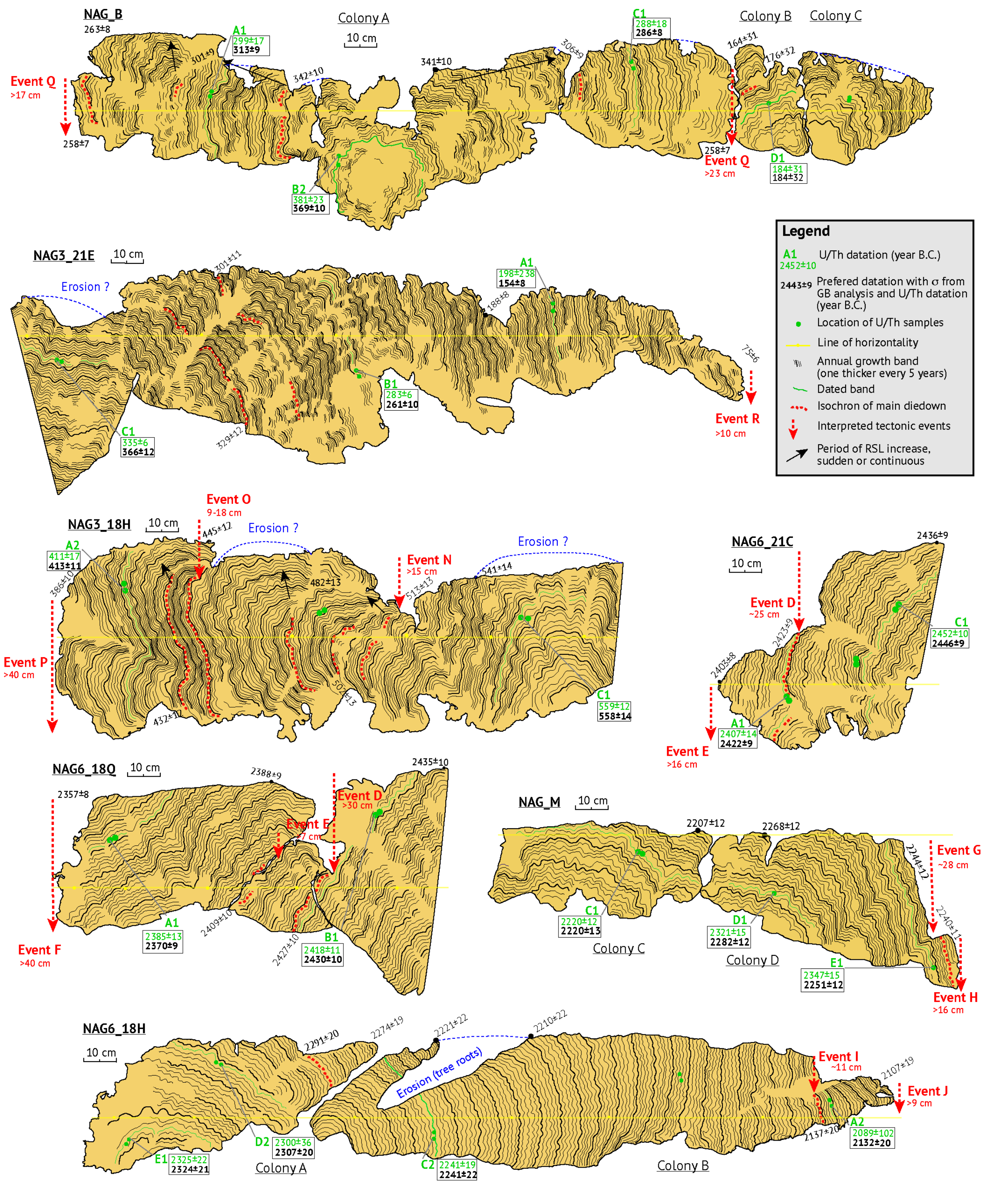

Figure S11. Interpreted slabs of sliced microatolls from their imagery. Black lines are growth bands, each of them marks 1 year. Thicker black lines are growth bands every 5 years. Black arrows represent relative submergence periods. Black dots mark the beginning or end of a relative sea-level (RSL) increase or decrease period. Age uncertainty is related to U/Th dating and growth band counting. Red dashed arrows show main interpreted RSL drops. Green lines are dated growth bands. Dashed blue lines show inferred significant erosion. GB = Growth Band.

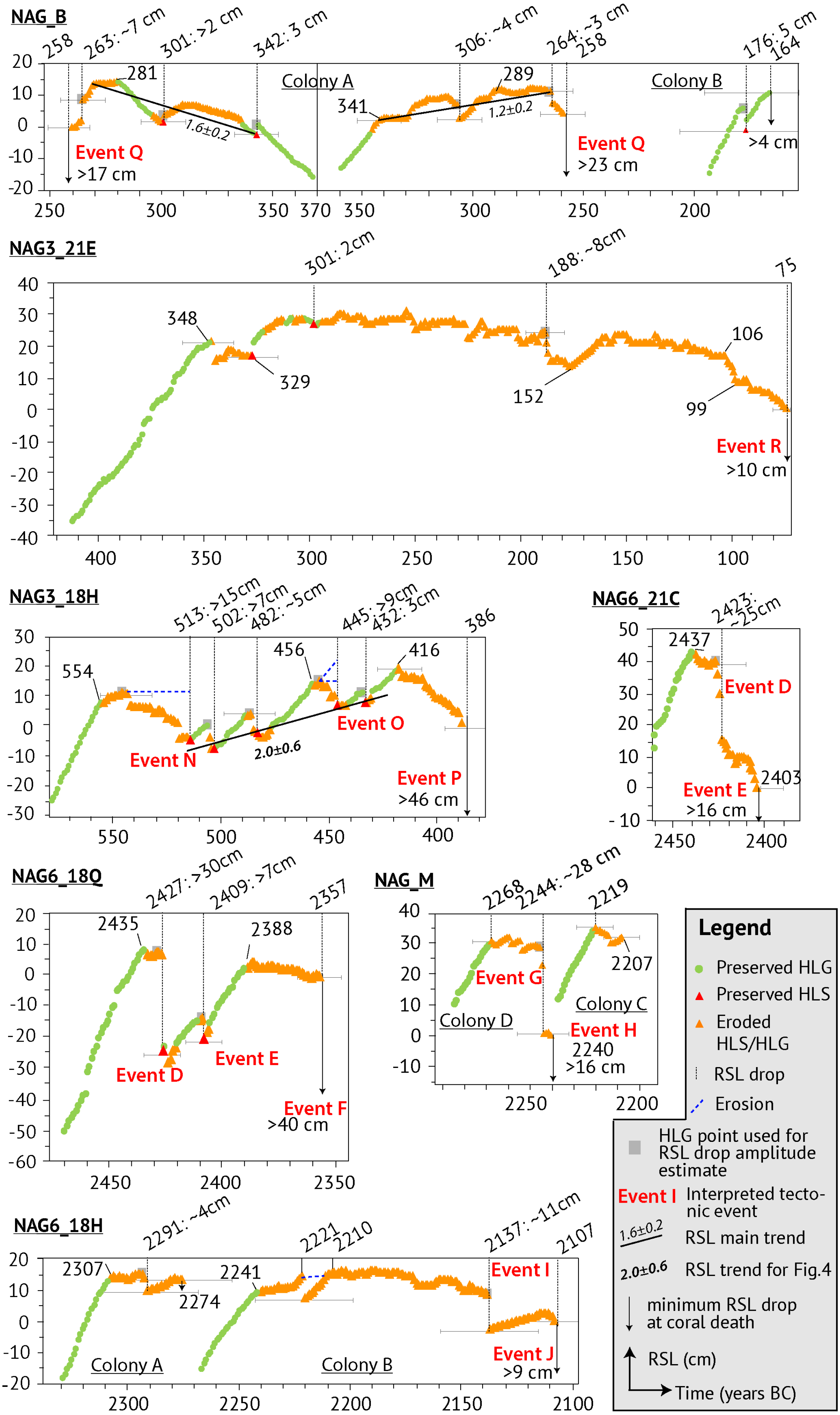
NAG_B
258
263: ~7 cm
301: >2 cm
342: 3 cm
306: ~4 cm
264: ~3 cm
258
176: 5 cm
164
281
Colony A
341
289
Colony B
1.6±0.2
1.2±0.2
Event Q
>17 cm
Event Q
>23 cm
>4 cm
NAG3_21E
301: 2cm
188: ~8cm
75
348
329
106
152
99
Event R
>10 cm
NAG3_18H
513: >15cm
502: >7cm
482: ~5cm
445: >9cm
432: 3cm
386
554
456
416
Event N
2.0±0.6
Event O
Event P
>46 cm
NAG6_21C
2423: ~25cm
2437
Event D
Event E
>16 cm
2403
NAG6_18Q
2427: >30cm
2409: >7cm
2357
2435
2388
Event D
Event E
Event F
>40 cm
NAG_M
2268
2244: ~28 cm
2219
Event G
2207
Colony C
Colony D
Event H
2240
>16 cm
NAG6_18H
2291: ~4cm
2221
2210
2137: ~11cm
2107
2307
2274
2241
Event I
Colony A
Colony B
Event J
>9 cm
Legend
Preserved HLG
Preserved HLS
Eroded HLS/HLG
RSL drop
Erosion
HLG point used for RSL drop amplitude estimate
Event I Interpreted tectonic event
1.6±0.2 RSL main trend
2.0±0.6 RSL trend for Fig.4
minimum RSL drop at coral death
RSL (cm)
Time (years BC)

Figure S12. Combination of all Highest Level of Survival (HLS) curves of our study. Uncertainty on ages, indicated by gray lines and related to U/Th dating and growth band counting, are shown in Fig. S11. Black arrows with amplitude show the sudden RSL drops that can explain the death of the microatoll. Other ages are indicated with continuous black line for the curve description. Dashed black line marks sudden drops of the RSL with amplitude in cm, with an uncertainty of ±1 cm. RSL rates in NAG_B are from Zach-1 method.

Text S2:

a) Detailed description of NAG_B site, slice and HLS

The NAG_B microatoll has an altitude of 0.15m below the WGS 84 ellipsoid. It is surrounded by many other fossil corals lying at comparable elevation. NAG_B area presents about twenty microatolls up to 100m away from the shoreline, with an average diameter of ~2m. Although they are partially buried, they mainly present a flat top surface or a cup shape (Figs. S11 and Section 1). We sampled a 2.5 m long slab of NAG_B slice along a diameter. The slab is made of three colonies (A, B, C, Fig. S11). The main one A is the central part of the microatoll, and two others (B and C) are external. The main coral started to grow in every direction. Very well-preserved patches of coralites were found at the base of the coral indicating that it was overturned at the onset of its growth. This is also revealed by the X-Ray imagery as the growth bands are concentric (Section 1, Fig. S2).

We collected by pairs and dated four samples in four growth bands along the slab (Fig. S11). Three on colony A: one in the eastern part of the central core (NAGB_A), one in the central part (NAGB_B) and one in the western part (NAGB_C); and one on colony B (NAGB_D). Four U/Th ages were obtained (Table S2). The samples NAGB_A1, NAGB_B2, NAGB_C1 in the eastern part, central ball-shaped part and western part of colony A are 299±17, 381±23 and 288±18 years B.C., respectively. NAGB_D1 sampled on colony B is 184±31 years B.C.
From the central overturned part of the colony, the left part of the coral developed upward growth at a rate of 6.5±0.2 mm/year during 25 years before reaching a HLS in 342±10 years B.C. (Fig. S12). It then grew laterally for 78 years and recorded a relative sea-level increase at rate of 1.6±0.2 mm/year. In 301±9 years B.C., it recorded a brief emergence event of at least 2 cm in amplitude and a second one later at 263±8 B.C. with an amplitude of 7 cm.
The right part of the colony A recorded similar RSL signal (Fig. S11). Mean submergence trends are observed between 341±10 and 265±8 years B.C. with a rate of 1.2±0.2 mm/year. We document two brief emergence events; the first one is of 4 cm at 306±9 years B.C.; the second one of 3 cm of amplitude was recorded at around 264±8 years B.C.. Both radii show a death of the coral due to a sudden sea-level fall of at least 23cm, at 258±7 years B.C. (Fig. S12).
The small coral against the main part of NAG_B coral is made of two small colonies, B and C (Fig. S11). Colony B is younger than colony A as it recorded RSL changes between 192±32 and 165±31 years B.C. The natural growth rate of the colony is of 13.7±0.4 mm/year, which is higher than for NAG_B main coral. We recorded a die-down of 5 cm of amplitude 176±32 years B.C.. During the following 12 years, the microatoll grew freely at a rate of 9.8±1.2 mm/year before it died completely at 164±31 years B.C..

b) Detailed description of NAG_M site, slice and HLS

The NAG_M coral has a diameter of 4 m. According to RTK-GPS measurements, it has a maximum altitude of 0.03, but we found its external part buried below ~15 cm of sand (Table S3). It is located in one of the main streams of the mangrove. We observed other microatolls next to NAG_M coral, and most of them are partially buried and have an average diameter of ~2.5 m. They mainly present a flat top surface. The cut slab of NAG_M is composed by two main blocks C and D and none was overturned (Fig. S11). Three samples were collected by pairs and dated on the different blocks (NAGM_C on colony C and NAGM_D and NAGM_E on colony D; Fig. S11, Table S2). An age of

2220±12 years B.C. (NAGM_C1) and two ages of 2321±15 and 2347±15 years B.C. were obtained on the growth band of the block D (NAGM_D1 and NAGM_E1) (Fig. S11).

The coral stratigraphy of colony C shows that it started to grow upward 2236±13 years B.C., at a growth rate of 13.5±0.4 mm/year (Table S4) until 2219±13 years B.C., and recorded a slight apparent RSL decrease of -3.4±1.4 mm/year during the 13 following years.
The second colony D began to grow upward at 2285±13 years B.C. at a rate of 12.1±0.4 mm/year, comparable to colony C, until 2268±12 years B.C. (Fig. S12). It then recorded a slight apparent relative sea-level decrease at a rate of -1.0±0.6 mm/year before recording a sea-level decrease of around 28 cm at 2244±12 years B.C.. After this event, only the lowest part of the microatoll survived four years before a second sudden sea-level decrease at 2240±11 years B.C.. After this sudden sea-level decrease, the colony completely died and only a minimum value of the sea-level drop of 16 cm can be estimated.

c) Detailed description of NAG6_18H sampling site, slice and HLS

NAG6_18H microatoll is a hatshape coral with a small external lower lobe, located in the mangrove area. It has an altitude of 0.18 m. It is partially eroded: it has a maximum size of 2.8 m and the length of the slice is of 2.6 m. The microatoll is composed of two colonies, colony A and colony B, which is the largest and the main one (Fig. S11).
We sampled and dated up to 4 growth bands, 2 on colony A and 2 on colony B. Samples NAG6_18H_E1 and NAG6_18H_D2 on colony A are dated at 2325±22 and 2300±36 years B.C., respectively. Samples NAG6_18H_A2 and NAG6_18H_C2 on colony B are dated at 2089±102 and 2241±19 years B.C., respectively.

The natural growth rate of colony A is of 15.9±0.6 mm/year. Starting from 2307±21 years B.C., the colony recorded an apparent slight RSL increase of 0.7±0.6 mm/year. After a sea-level drop of around 4 cm in amplitude at 2291±20 years B.C., the coral underwent a period of apparent RSL increase at a rate of 2.7±0.4 mm/year until 2275±19 years B.C. The end of the colony A record may not correspond to the exact date of its death as the oldest edge of the colony seems slightly eroded.
Colony B started to grow few years later at an initial growth rate of 9.6±0.2 mm/year. It recorded a period of apparent RSL increase of 2.0±0.4 mm/year starting from 2240±22 years B.C. The colony shows on its top surface bioerosion related to mangrove roots, which alters its RSL records from 2221±22 to 2210±52 years B.C. Between 2210±22 and 2138±20 years B.C., colony B underwent an apparent RSL decrease of -0.9±0.1 mm/year, before a sudden drop of the RSL of around 11 cm at 2137±20 years B.C. Afterwards, we observe a period of 30 years of relative submergence at a rate of 1.8±0.6 mm/year. The record stops at 2108±19 years B.C., after which the colony likely died after a RSL drop of at least 9 cm (Fig. S12).

d) Detailed description of NAG6_18Q sampling site, slice and HLS

In the mangrove area, NAG6_18Q microatoll is a 5 m-wide hatshape coral with an altitude of 0.03 m (Fig. S11). It is made of several colonies, and we only collected its external part as the slice we could sample is 1.1 m long. We sampled by pairs two growth bands for dating, which samples NAG6_18Q_A1 and NAG6_18Q_B1 are dated at 2385±13 and 2418±11 years B.C., respectively (Fig. S11, Table S2).
NAG6_18Q has an initial growth rate of 17.9±0.7 mm/year. We observe a die-down of at least 30 cm at 2427±10 years B.C. Following this die-down, the stratigraphy of the coral shows a period of growth of 7.7±1.2 mm/year interrupted by a sudden RSL drop of at least 7 cm, at 2409±10 years B.C.. It then recorded an apparent relative submergence at a rate of 11.5±0.4 mm/year until 2388±9 years B.C.. Afterwards, the coral underwent a period of apparent RSL decrease at a rate of -1.4±0.2 mm/year until its death at 2357±8 years B.C., which is marked by a strong RSL drop with a minimum amplitude of 40 cm.

e) Detailed description of NAG6_21C sampling site, slice and HLS

In the mangrove area, NAG6_21C microatoll was collected close from NAG6_18Q coral. It has a slightly lower altitude (0.03 m below the WGS 84 ellipsoid, Table S4), and it also is a hatshape coral with a lower external lobe. The coral was partially eroded, and its maximum size is of 1.3m. The slice we collected is 0.7m long. Beyond the three growth bands we sampled, two of them were dated as samples NAG6_21C_A1 and NAG6_21C_C1 are dated at 2407±14 and 2452±10 years B.C., respectively (Fig. S11).
NAG6_21C has a natural growth rate of 14.0±0.8 mm/year. It recorded HLS variations at 2436±9 years B.C. at the soonest, with a period of apparent RSL decrease of -1.8±1.4 mm/year. At 2423±9 years B.C., we observe a strong RSL drop of around 25 cm, followed by a period of slight apparent relative emergence at a rate of 5.3±1.4 mm/year. This period stops at 2403±8 years B.C., where the death of the coral is marked by a second RSL drop with an amplitude of at least 16 cm (Figs. S11 and S12).

f) Detailed description of NAG3_18H sampling site, slice and HLS

Located in the bay area, NAG3_18H microatoll is a clear cupshape surrounded by other cupshape corals, and it has an altitude of 0.50 m below the WGS 84 ellipsoid. Several parts of the coral may have been buried and/partially exposed to erosion. The colony has a diameter of 2.5m, and the slice we extracted has a length of 1.5 m. We collected by pairs 3 samples of growth bands. The most internal one, NAG3_18H_C1, is dated at 559±12 years B.C., and the most external one, NAG3_18H_A2, is dated at 411±17 years B.C..
NAG3_18H natural growth rate is of 14.7±0.2 mm/year. The initial growth of the coral might stop at 551±14, but we suspect the upper part of the coral has been eroded since, establishing the first record of the HLS or HLG at 513±13 years B.C. at the later (Fig. S12). Between 541±14 and 513±13 years B.C., the coral underwent a period of apparent relative emergence with a rate of -4.2±0.8 mm/year. We infer a drop of the RSL of at least 15 cm (considering the highest eroded HLG/HLS band prior to the diedown, see Ref[5]), dated at 513±13 years B.C.. Between 513±13 and 456±12 years B.C, the coral recorded three periods of growth with a rate of ~7.0 mm/year, interrupted at 502±13 and at 482±13 years B.C. by RSL drops of at least 7 cm and around 5 cm, respectively. Between 456±12 and 445±12 years B.C., the coral upper surface may have undergone another erosion, smaller in amplitude (Fig. S11). If the apparent RSL increase period last until 445±12 years B.C., the coral would have recorded a RSL drop of 18 cm (instead of 9 cm, considering the highest eroded HLS/HLG prior to the diedown[5], Fig. S12). Between 445±12 and 417±11 years B.C., the microatoll recorded a slight apparent relative submergence of 4.0±0.6 mm/year, interrupted at 432±12 years B.C. by a RSL drop of 3 cm. Between 416±11 and 387±10 years B.C., we observe an apparent RSL decrease of -5.6±0.4 mm/year. The death of the coral may correspond to a large RSL drop of at least 46 cm at 386±10 years B.C..
With up to five HLS growth band preserved from erosion after a diedown, we can estimate a relative submergence trend of 2.0±0.6 mm/year between 513±13 and 417±11 years B.C.. Such submergence rate extended until the death of the coral would imply a RSL drop of 75 cm at 386±10 years.

g) Detailed description of NAG3_21E sampling site, slice and HLS

NAG3_21E is a large hatshape microatoll located in the bay area. It has an altitude of 0.82 m below the WGS 84 ellipsoid. Its diameter is of 4.5m, and the slice we sampled is 2.3 m long. We collected samples by pairs for dating on three growth bands, with an age of 335±6, 283±6 and 198±238 years B.C. from the most internal (NAG3_21E_C1) to the most external one (NAG3_21E_A1, Fig. S11).
The internal part of the coral is lower than its surrounding parts. However, the coral stratigraphy may show an erosion until at least 348±12 years B.C. (Fig. S11). The natural growth rate of the coral is of 9.2±0.2 mm/year. Between 347±12 and 302±11 years B.C., it recorded an apparent RSL increase of 3.5±0.2 mm/year. It then recorded a period of apparent RSL stability of -0.6±0.1

mm/year between 301±11 and 106±7 years B.C. This period was interrupted by at least small die-down at 301±11 years B.C. with a minimum amplitude of 2 cm. A larger RSL drop is observed at 188±8 years B.C. with an amplitude of around 8 cm, followed by a small period of apparent RSL increase of 3.1±0.4 mm/year of 36 years. Finally, between 106±7 and 76±6 years B.C., we observe two apparent relative emergence periods before and after 99±7 years B.C. at a rate of -12.4±1.6 mm/year and -3.6±0.4 mm/year, respectively. The coral died at 75±6 years B.C., with a RSL drop of at least 10 cm of amplitude.

Table S4. Relative emergence and submergence rates from the relative sea-level variations recorded by our microatolls samples in mm/year (see Method[5-8]). Positive and negative values indicate relative submergence and emergence, respectively.

Slice	Period (years B.C.)	All HLG points	Zach-1 (All points)	Zach-2 (All HLS)	Zach-3 (non-eroded HLS)	Meltzner (highest points prior to diedowns)
NAG3_18H	577-554	14.7±0.2	14.7±0.2			
	541-514		-4.2±0.8	-4.2±0.8		
	502-485		7.4±0.5	6.9±0.4		
	482-456		6.7±0.4	1.4±3.2		
	445-433		3.7±0.8	-0.1±0.8		
	432-417		7.0±0.4	5.3±0.2		
	416-387		-5.6±0.4	-5.6±0.4		
	445-417		4.0±0.6	1.3±0.4	~0.9	
	**513-417**		2.2±0.2	2.4±0.6	**2.0±0.6**	**1.9±0.7**
NAG3_21E	414-348	9.2±0.2	9.2±0.2			
	347-302		3.5±0.2	3.7±0.3		
	301-106		-0.6±0.1	-0.6±0.1		
	188-152		3.1±0.4	3.1±0.4		
	106-99		-12.4±1.6	-12.4±1.6		
	99-76		-3.6±0.4	-3.6±0.4		
NAG_B (left)	369-343	6.5±0.2	6.5±0.2			
	342-264		1.6±0.2	2.0±0.4	~0.9	**1.1±0.2**
NAG_B (right)	359-341	8.2±0.2	8.9±1.5	10.4±1.0		
	341-265		1.2±0.2	1.2±0.2		~0.9
NAG_B (colony B)	192-177	13.7±0.4	13.7±0.4			

Slice	Period (years B.C.)	All HLG points	Zach-1 (All points)	Zach-2 (All HLS)	Zach-3 (non-eroded HLS)	Meltzner (highest points prior to diedowns)
NAG6_18H (colony A)	2328-2307	15.9±0.6	15.9±0.6			
	2307-2292		0.7±0.6	0.7±0.6		
	2291-2275		2.7±0.4	2.7±0.4		
	2307-2275		-6.2±0.6	-6.2±0.6		
NAG6_18H (colony B)	2266-2241	9.6±0.2	9.6±0.2			
	2240-2221		2.0±0.4	2.0±0.4		
	2210-2138		-0.9±0.1	-0.9±0.1		
	2137-2108		1.8±0.6	1.8±0.6		
NAG_M (colony D)	2285-2269	12.1±0.4	12.1±0.4			
	2268-2246		-1.0±0.6	-1.0±0.6		
NAG_M (colony C)	2236-2220	13.5±0.4	13.5±0.4			
	2219-2207		-3.4±1.4	-3.4±1.4		
NAG6_18Q	2470-2435	17.9±0.7	17.9±0.7			
	2427-2410		7.7±1.2	8.0±2.4		
	2409-2388		11.5±0.4	11.6±0.6		
	2388-2358		-1.4±0.2	-1.4±0.2		
NAG6_21C	2458-2437	14.0±0.8	14.0±0.8			
	2436-2426		-1.8±1.4	-1.8±1.4		
	2423-2404		-5.3±1.4	-5.3±1.4		

Note. We compare Zach_1, Zach-2, Zach_3 and Meltzner methods (see Method). All methods give comparable results. The bold values indicate well-constrained trends. Other values are partially inferred from eroded HLG/HLS and/or HLG points; they are referred in the text as "apparent" RSL decrease or increase. Underlined value from NAG3_18H coral is used to reconstruct the RSL at a larger time scale in Fig. 4. Periods in gray are initial growth period, that correspond to HLG years and that will not be considered further in this study.

Table S5. Weighted average dates of presumed events. Amplitude of each event is inferred from slice analysis and/or RSL reconstruction in Figs. 3 and 4 (see Method). Letter with an apostrophe denote subsidence, while those without denote uplift. Amplitude of events identified only in the slices are given in range (Figs. S12 and 3). Each event is assigned with a confidence index from 1 to 6. A confidence index of 6 indicates the best-reliable event (see Method).

Name of Events	Dated coral blocks	Name of Slices	Age (year)	All-data age average (year)	Amplitude (cm)	Confi dence index	Supporting evidences
A	NAG2_18G		2855±40 BC	2855±40 BC	75±22	3	Death of NAG2_18G
B	NAG1_18B		2739±80 BC	2739±80 BC	25±24	3	Death of NAG1_18B & corals at same elevation
C	NAG7_18A		2482±41 BC	2482±41 BC	45±12	4	Death of NAG7_18A & corals at same elevation
D		NAG6_18Q<br>NAG6_21C	2427±10 BC<br>2423±9 BC	2425±7 BC	>30	6	Slab analysis (preserved growth bands)
E		NAG6_18Q<br>NAG6_21C	2409±10 BC<br>2403±8 BC	2405±6 BC	>16	6	Slab analysis (preserved growth bands)
F		NAG6_18Q	2357±8 BC	2357±8 BC	>40	3	Death of NAG6_18Q
A'				2307-2357	> -35	4	Difference of elevation between group of corals (Fig. 3)
G	NAG6_21A	NAG_M	2244±12 BC<br>2252±83 BC	2244±12 BC	~28	5	Slab Analysis (eroded growth bands)
H		NAG_M	2240±11 BC	2240±11 BC	>16	3	Death of NAG_M
I	NAG5_21A	NAG6_18H	2137±20 BC<br>2128±330 BC	2137±20 BC	~11	5	Slab analysis (eroded growth bands)
J		NAG6_18H	2107±19 BC	2107±19 BC	>9-58±18	3	Death of NAG6_18H
K	NAG5_18B		1970±65 BC	1970±65 BC	136±41	4	Death of NAG5_18B & corals at same elevation
L	GIANT_21		1324±23 BC	1324±23 BC	166±60	3	Death of one colony in GIANT

Name of Events	Dated coral blocks	Name of Slices	Age (year)	All-data age average (year)	Amplitude (cm)	Confidence index	Supporting evidences
M	NAG3_18C		627±212 BC	627±212 BC	15±43	3	Death of NAG3_18C & corals at same elevation
N		NAG3_18H	513±13 BC	518±13 BC	>15	5	Slab Analysis (eroded growth bands)
O		NAG3_18H<br>NAG3_18H	445±12 BC<br>386±10 BC	445±12 BC	9-18	5	Slab analysis (eroded growth bands)
P	GIANT_18	 NAG3_18H	367±8 BC<br>386±10 BC	374±6 BC	>46-75	4	Death of one colony in GIANT and death of NAG3_18H
Q	NAG3_18B	 NAG_B	253±115 BC<br>258±7 BC	256±6 BC	>23-40	4	Death of NAG_B and NAG3_18B, & corals at same elevation
R		NAG3_21E	75±6 BC	75±6 BC	>10-262±75	3	Death of NAG3_21E
S	YAMA_2		1157±92 AD	1157±92 AD	93±79	2	Observed coral in Yamaguchi (2016)
T	YAMA_1		790±97 AD	790±97 AD	50±65	1	Observed coral in Yamaguchi (2016)
U	C11		1520±430 AD (presumed)	1771 AD (Meiwa)	23±21	1	Hypothesis

Supplementary Information S3: Elastic modelling

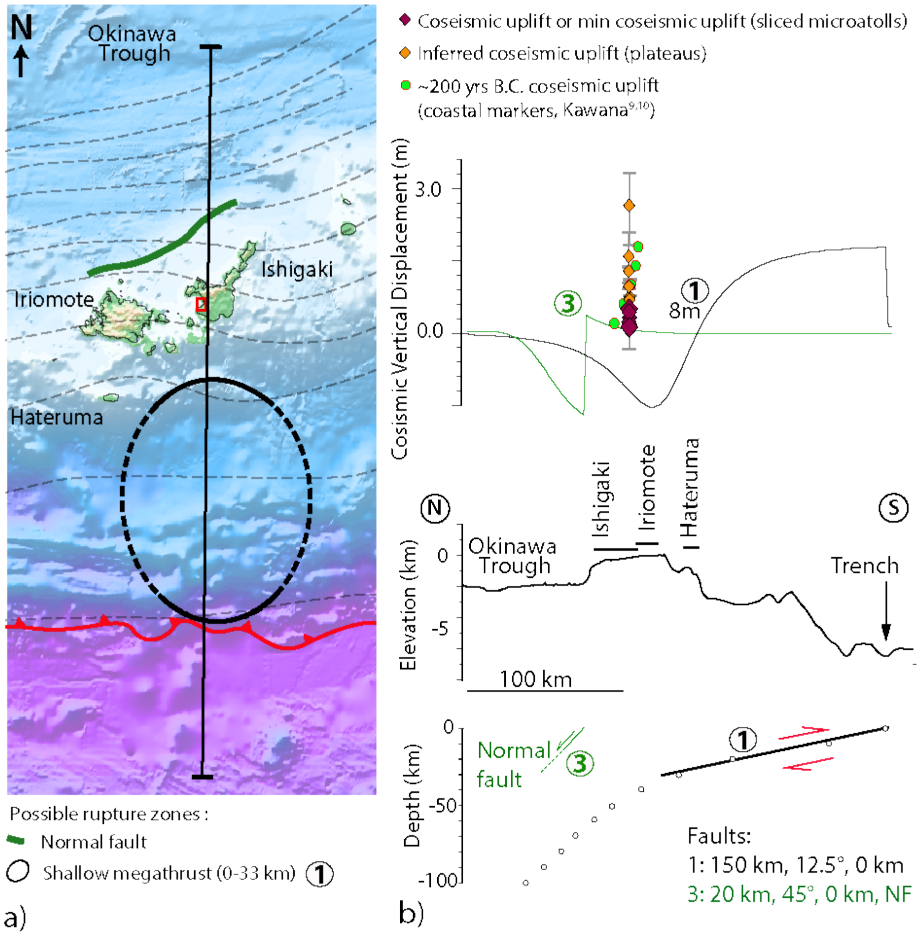

Figure S13. Elastic models of rupture sources in the tectonic setting of Ishigaki area. a) Map of the potential rupture zones investigated here. Slab depth, continuous black line, red line with arrows and doted gray lines as in Fig. 5. Rupture zones lengths are uncertain, we estimate a maximum length of 300km along the southern Ryukyus (see text, Fig. 1). Panel produced using the Generic Mapping Tool software (https://www.generic-mapping-tools.org/) b) 2D Elastic models of the coseismic deformations in Nagura. All models are performed in an elastic half space λ = μ = 32 GPa. Green curve: vertical deformation at the surface promoted by slips on a hypothetic normal fault at the southern border of Okinawa Trough. Black curve: vertical deformation at the surface promoted by slips on the shallow part of the megathrust, between 0 and 33 km depth.

Text related to Fig. S13

Coseismic deformation induced by normal faulting and shallow megathrust ruptures
We attempted to reproduce the coseismic deformation we observed with slip induced by normal faulting at the southern margin of the Okinawa through (Fig. S13). We used a 20 km-wide dislocation dipping northward by 45° with a slip of 3 m (implying a magnitude 7.7 for a length of 100 km). Given the size and magnitude, such a dislocation would maximize any normal faulting event along the Okinawa through north of Ishigaki and Iriomote (Fig. S13). We observe that dislocation on a normal fault in the Okinawa Trough (fault 3) generates a local uplift that rapidly decrease towards the trench, and that is negligible in our study sites.
We also modelled surface deformation linked to slip on the shallowest portion of the megathrust (fault 1), which induced subsidence rather than uplift along the archipelago. We consider that such model also discards splay faults as possible origin of the coseismic deformations we document, as they are most likely connected in deep with the megathrust (Fig. S13). Dislocation on deeper part of the megathrust is therefore required.

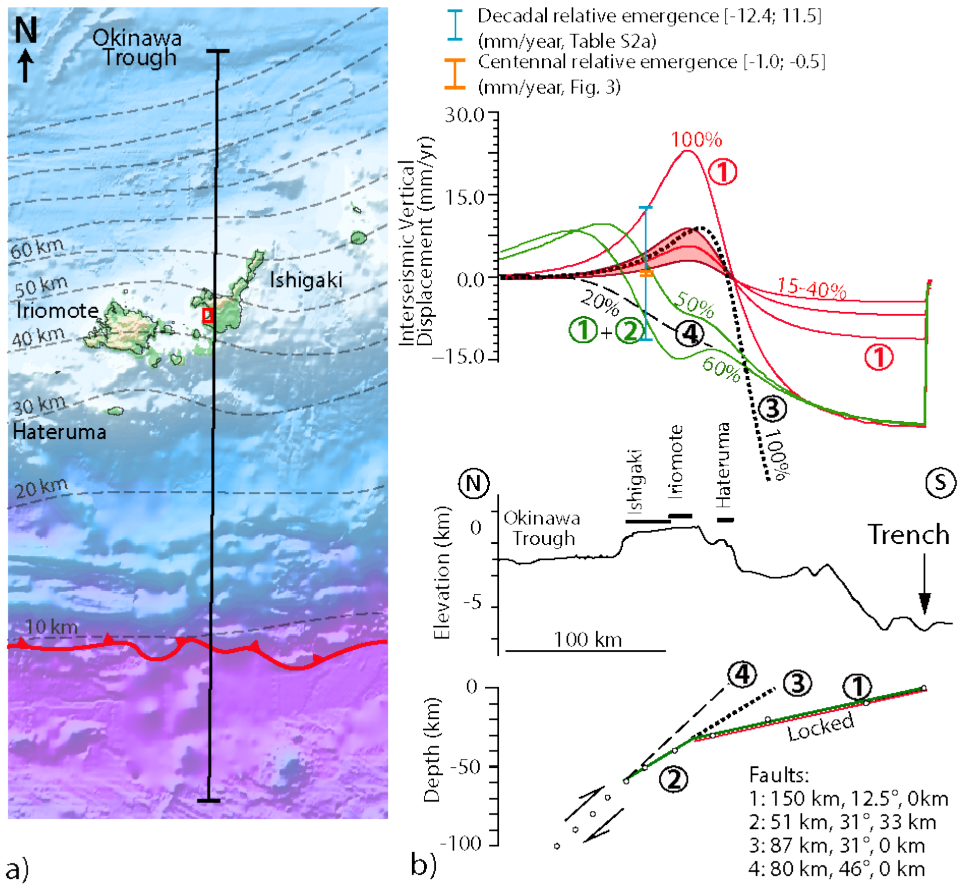

Figure S14. Elastic models of interseismic RSL changes in Nagura. a) Tectonic setting of the area as in Fig. S13. Panel produced using the Generic Mapping Tool software (https://www.generic-mapping-tools.org/) b) 2D modeled interseismic deformation. All models are performed in an elastic half space with $\lambda = \mu = 32$ GPa. Red area: vertical deformation rate promoted by backslip with seismic coupling between 15% and 40% along fault 1 between 0 and 35 km depth. A coupling of 100% corresponds to an annual slip of 12 cm as the convergence rate is estimated at 12 cm/year[11]. Decadal emergence trends are inferred from slice analysis (Table S4) and refer to either apparent or well-constrained rates.

Text related to Fig. S14

Interseismic deformation modelling

The interseismic deformations estimated from the coral slices analysis are modeled in Fig. S14. Apparent interseismic decadal RSL changes at rate between 0 and 11.5 mm/year (Table S4) can be accounted for by strain accumulation on the shallowest part of the megathrust coupled at 15 to 40% (~25% in average, or 100% depending on the dislocation models). Apparent decadal decrease of the RSL at rate between 0 to -12.4 mm/year (Table S4) occur when the plate interface is locked deeper (e.g. strain accumulation on F1+F2) by at least 50 %. As we observed alternation of subsidence and uplift during the interseismic period in the record from coral slices, the strain can thus accumulate on the whole interface until 60 km depth or only on shallower asperities.
Centennial RSL decrease trends inferred from Fig. 3 would rather indicate either a weak coupling on the shallowest part of the megathrust, either a coupling of ~50% between 0 and 60km depth.

Supplementary References

1. Hiess, J., Condon, D. J., McLean, N. & Noble, S. R. ^{238}U/ ^{235}U Systematics in Terrestrial Uranium-Bearing Minerals. *Science* **335**, 1610–1614 (2012).
2. Cheng, H. *et al.* Improvements in 230Th dating, 230Th and 234U half-life values, and U–Th isotopic measurements by multi-collector inductively coupled plasma mass spectrometry. *Earth and Planetary Science Letters* **371–372**, 82–91 (2013).
3. Shen, C.-C. *et al.* Variation of initial 230Th/232Th and limits of high precision U–Th dating of shallow-water corals. *Geochimica et Cosmochimica Acta* **72**, 4201–4223 (2008).
4. Yamaguchi, T. A Review of Coral Studies of the Ryukyu Island Arc to Reconstruct Its Long-Term Landscape History. in *Coral Reef Science: Strategy for Ecosystem Symbiosis and Coexistence with Humans under Multiple Stresses* (ed. Kayanne, H.) 55–63 (Springer Japan, Tokyo, 2016). doi:10.1007/978-4-431-54364-0_4.
5. Meltzner, A. J., Sieh, K., Chiang, H. W., Shen, C. C., Suwargadi, B. W., Natawidjaja, D. H., ... & Galetzka, J. (2010). Coral evidence for earthquake recurrence and an AD 1390–1455 cluster at the south end of the 2004 Aceh–Andaman rupture. *Journal of Geophysical Research: Solid Earth*, *115*(B10).
6. Zachariasen, J. A. Paleoseismology and Paleogeodesy of the Sumatran Subduction Zone: A Study of Vertical Deformation Using Coral Microatolls. (California Institute of Technology, 1998). doi:10.7907/YYWJ-R395.
7. Zachariasen, J., Sieh, K., Taylor, F. W., Edwards, R. L. & Hantoro, W. S. Submergence and uplift associated with the giant 1833 Sumatran subduction earthquake: Evidence from coral microatolls. *Journal of Geophysical Research: Solid Earth* **104**, 895–919 (1999).
8. Zachariasen, J., Sieh, K., Taylor, F. W. & Hantoro, W. S. Modern Vertical Deformation above the Sumatran Subduction Zone: Paleogeodetic Insights from Coral Microatolls. *Bulletin of the Seismological Society of America* **90**, 897–913 (2000).
9. Kawana, T. Holocene crustal movements in and around the Sekisei Lagoon area of the South Ryukyu Islands. *The Earth Monthly* **9**, 129–134 (1987).
10. Kawana, T. Quaternary crustal movement in the Ryukyu Islands. *Earth Monthly* **11**, 618–30 (1989).
11. Chen, Horng-Yue, et al. "Strain partitioning in the southern ryukyu margin revealed by seafloor geodetic and seismological observations." Geophysical Research Letters 49.6 (2022): e2022GL098218.
